# Screening and identification of the dominant antigens of the African swine fever virus

**DOI:** 10.3389/fvets.2023.1175701

**Published:** 2023-05-04

**Authors:** Zhaoyang Xu, Yifan Hu, Junbo Li, Ancheng Wang, Xin Meng, Lingchao Chen, Jianchao Wei, Wu Tong, Ning Kong, Lingxue Yu, Hai Yu, Tongling Shan, Guangzhi Tong, Guihua Wang, Hao Zheng

**Affiliations:** ^1^Shanghai Veterinary Research Institute of Chinese Academy of Agricultural Sciences, Shanghai, China; ^2^College of Veterinary Medicine of Shandong Agricultural University, Tai'an, China; ^3^College of Wildlife and Protected Area, Northeast Forestry University, Harbin, China; ^4^Jiangsu Co-Innovation Center for the Prevention and Control of Important Animal Infectious Disease and Zoonosis, Yangzhou University, Yangzhou, China

**Keywords:** African swine fever virus, dominant antigen, p30 protein, p54 protein, p22 protein

## Abstract

African swine fever is a highly lethal contagious disease of pigs for which there is no vaccine. Its causative agent African swine fever virus (ASFV) is a highly complex enveloped DNA virus encoding more than 150 open reading frames. The antigenicity of ASFV is still unclear at present. In this study, 35 proteins of ASFV were expressed by *Escherichia coli*, and ELISA was developed for the detection of antibodies against these proteins. p30, p54, and p22 were presented as the major antigens of ASFV, positively reacting with all five clinical ASFV-positive pig sera, and 10 pig sera experimentally infected by ASFV. Five proteins (pB475L, pC129R, pE199L, pE184L, and pK145R) reacted well with ASFV-positive sera. The p30 induced a rapid and strong antibody immune response during ASFV infection. These results will promote the development of subunit vaccines and serum diagnostic methods against ASFV.

## 1. Introduction

African swine fever, caused by the African swine fever virus (ASFV), is a highly lethal contagious disease in domestic pigs and wild boars. It affects swine of all breeds and ages, giving rise to a variety of clinical signs and lesions, from acute hemorrhagic fever with high mortality to chronic infection with skin ulcers and joint swelling, and causes serious economic consequences in the pig industry (1). Therefore, it is included in the list of diseases obliged declaration to the World Organization for Animal Health (OIE).

African swine fever (ASF) was first described in 1921 in Kenya and soon became enzootic throughout sub-Saharan Africa. It first crossed continents to Portugal in 1957 and from there to other European and Latin American countries, from where the disease was successfully eradicated in the mid-1990s, except for Sardinia (2). A new transmission era started when ASF was introduced into Georgia from Southeast Africa in 2007. Subsequently, it spread progressively across the Eurasian continent (2). Currently, most countries in Central and Eastern Europe and East and Southeast Asia have reported ASF cases, and it has even appeared in several Western European countries (3, 4). ASF was first reported in China in August 2018 (5). Within 6 months, acute ASF with almost 100% mortality had spread throughout most provinces in China and caused devastation to thousands of Chinese pig farms (4, 6). After 2 years of major efforts, acute ASF has been controlled to a great extent. In early 2021, however, ASFV with low virulence was found on some Chinese pig farms (7). Then, low-virulent isolates became predominant in the ASFV epidemic in China. Currently, ASFV is classified as a Class I infectious pathogen, and ASF has become the number one swine disease in China (6).

African swine fever virus (ASFV) is the sole characterized member of Asfarviridae and has a linear double-stranded DNA genome of 170–194 kbp encoding 150–170 predicted open reading frames (ORFs) (3). The differences in viral genome length and gene number predominantly originate from the loss or gain of genes in multigene families at the genome termini. A total of 94 viral proteins were expressed in cell lines infected with ASFV (8). However, the expression profiles of ASFV proteins in different cell lines varied markedly. A total of 68 viral proteins have been identified in mature virions (9). Many viral proteins, especially those containing transmembrane domains, have an unknown function. Characterization of viral antigens is important for developing ASF vaccine and serodiagnostic methods against ASFV. Several viral proteins such as p30, p54, p72, and CD2v are the major antigens of ASFV, and subunit vaccines made from these proteins provide partial protection against virulent ASFV challenge (10). Pigs immunized with mixed DNA vaccines or recombinant vaccinia virus expressing these proteins are partially protected against virulent ASFV (11, 12). Immunization with a DNA expression library containing >4,000 plasmids cloned with ASFV genome fragments clearly improved the protection against ASFV (13). These results suggested that many protective antigens were unexplored.

In this study, 35 structural and highly expressed ASFV proteins were prepared by a prokaryotic expression system. To evaluate the antigenicity of these proteins in ASFV infection, the levels of specific antibodies against these proteins in ASFV-positive pig sera were detected by ELISA.

## 2. Materials and methods

### 2.1. Sera

A total of 26 inactivated sera from pigs experimentally infected with the CD2v and UK gene-deleted ASFV vaccine candidate (ASFV-SY18-ΔCD2v/UK) were kindly provided by Dr. Sun at Harbin Veterinary Research Institute, Harbin, China. Among these, 10 sera from pigs infected with ASFV-SY18-ΔCD2v/UK at 49 days post-inoculation (dpi) were served as ASFV-positive sera (14). A total of 16 sera were collected from four pigs infected with ASFV-SY18-ΔCD2v/UK at 6, 10, 15, and 21 dpi. In total, five inactivated clinical sera were obtained from farm pigs that had recovered from ASFV infection and also served as ASFV-positive sera. A total of five sera as negative controls were collected from healthy pigs in 2017. The ASFV-positive or ASFV-negative sera were tested by the ID Vet ASFV p30 antibody detection kit and the Ingenasa ASFV p72 ELISA kit.

### 2.2. Construction of expression plasmids of ASFV genes

The genome of genotype II ASFV strain Pig/HLJ/2018 (MK333180) was used as a reference sequence. A total of 38 genes of ASFV including 32 major structural proteins and seven proteins highly expressed in cells were analyzed with Protean in the LaserGene (DNAStar, Madison, Wisconsin, USA) and the online software of ProtScale (URL), SignalP, and TMHMM. The signal peptide and transmembrane sequences were deleted from transmembrane proteins. When a protein was >500 amino acids in length, the major antigenic fragment with 200–300 amino acids was used for expression. After optimization with *Escherichia coli* codon, these sequences were synthesized by General Biol (Anhui, China) and cloned into the prokaryotic expression vector pCold I with the restriction enzymes *Nde*I/*Xba*I, respectively. All constructs were verified by DNA sequencing and transformed into competent *E. coli* strain BL21 (TaKaRa Biotechnology (Dalian), Dalian, China).

### 2.3. Protein purification

Transformed BL21 cells were grown overnight in Luria–Bertani (LB) liquid medium with ampicillin (100 μg/ml). The culture was diluted 1:100 in fresh LB medium and grown for 2.5 h. Expression of recombinant proteins was induced with 1 mM isopropyl β-d-1-thiogalactopyranoside (IPTG) for 16 h at 16°C, and cells were harvested by centrifugation at 5,000 rpm in an Eppendorf F-34-6-38 rotor for 10 min. Soluble proteins were purified using WorkBeads 40 Ni-NTA (Bio-Works, Upasala, Sweden). Insoluble proteins were purified after solubilization with urea. Cell pellets were resuspended in 10 ml 50 mM Tris/HCl, and lysozyme (1.25 mg) was added. The cells were lysed by five cycles of sonication and then centrifuged. The pellet was washed three times in 5 ml 50 mM Tris/HCl with 1% Triton X-100. Next, 8 M urea (2.5 ml) was added, and the mixture was sonicated until the pellet was dissolved. The suspension was centrifuged at 6,000 rpm in an Eppendorf F-34-6-38 rotor for 10 min. The denatured protein was purified with WorkBeads 40 Ni-NTA and then refolded by dialysis in PBS.

### 2.4. ELISA

ELISA plates (Corning Inc., Corning, New York, USA) were coated with ASFV recombinant proteins (100 μl per well) diluted to the appropriate concentrations (1 μg/ml) in coating buffer (50 mM sodium carbonate/bicarbonate buffer, pH 9.6) and incubated overnight at 4°C. The wells were washed three times with PBS plus 0.05% Tween 20 and blocked with PBS plus 5% milk (200 μl per well) at 37°C for 1 h. After blocking, plates were washed five times as mentioned above and incubated for 1 h at 37°C with pig sera diluted 1:100 in PBS plus 5% milk (100 μl per well). The plates were washed again five times and incubated with horseradish-peroxidase-conjugated anti-pig IgG antibody (Sigma–Aldrich, Saint Louis, Missouri, USA) diluted 1:4,000 (100 μl per well) for 1 h at 37°C. Finally, the plates were washed again and developed with TMB in the dark at room temperature for 15 min. After stopping the reaction with 2M H_2_SO_4_ (50 μl per well), the A450 was read on a Bio-Rad microplate reader (Bio-Rad, Hercules, California, USA).

### 2.5. Statistical analysis

Data were analyzed using GraphPad Prism 6.0 software. All data are presented as the mean ± standard deviation (SD). A *p*-value of <0.05 was considered statistically significant.

## 3. Results

### 3.1. Expression and purification of 35 ASFV proteins

The ASFV genome contains more than 150 ORFs, and the virion consists of nearly 70 proteins. To explore the antigenicity of ASFV in infected pigs, 38 viral genes (Table 1) were expressed by *E. coli*. Among these genes, *O61R* (p12 gene), *D117L* (p17 gene), and *K78R* (p10 gene) were fused together with linker sequences, and *I73R* was fused with *B169L*. The amino acid sequences of expressed ASFV proteins are presented in Supplementary material S1. After induction with IPTG, four proteins (p54, pA104R, p11.5, and pI73R-pB169L) were expressed in soluble form, and the other 31 proteins (p30, pH171R, pCP312R, pE199L, pC257L, pE120R, p72, p22, pI177L, pK196R, pE146L, pF317L, pC717R, pB475L, pB602L, pEP152R, pE248R, pE184L, pH240R, pM1249L, pB125R, pC129R, pK421R, pEP153R, pK205R, pK145R, p150, CD2v, p15, p34, and p12-p17-p10) were expressed in the form of inclusion bodies. As shown in Supplementary material S2, 35 proteins were purified with Ni-NTA and used to coat ELISA plates.

**Table 1:** ASFV proteins expressed by *E. coli*.

**Protein**	**Size (kDa)**	**Gene**	**Function**	**Structural role**
CD2v	41	*EP402R*	Evasion from host defense, regulating virulence	Outer envelope
p12	6.9	*O61R*	Virus entry	Outer envelope
pE120R	13.9	*E120R*	Morphogenesis	Capsid
p72	73.2	*B646L*	Morphogenesis	Capsid
pH240R	27.7	*H240R*	Regulating virulence	Capsid
pM1249L	145.3	*M1249L*	Morphogenesis and inhibiting type I interferon production	Capsid
p17	13.1	*D117L*	Morphogenesis	Inner envelope
p30	23.2	*CP204L*	Genome replication, virus entry	Inner envelope
p22	20.7	*KP177R*	Genome replication, regulating virulence	Inner envelope
p54	20	*E183L*	Virus structure and morphogenesis	Inner envelope
pE199L	22.7	*E199L*	Virus entry	Inner envelope
pE248R	27.4	*E248R*	Virus entry	Inner envelope
p150	181.2	*CP2475L*	Morphogenesis	Core shell
p15	17.9	*CP530R*	Morphogenesis	Core shell
p34	36.6	*CP2475L*	Morphogenesis	Core shell
pA104R	11.6	*A104R*	DNA-binding proteins and morphogenesis	Nucleoid
p10	8.4	*K78R*	DNA-binding proteins and morphogenesis	Nucleoid
p11.5	16.2	*A137R*	Regulating virulence	Virion
pI73R	8.4	*I73R*	Regulating transcription	Virion
pI177L	20.5	*I177L*	Regulating virulence, transmembrane domain	Virion
pF317L	36.8	*F317L*	Evasion from host defense, genome replication	Virion
pE184L	21.8	*E184L*	Regulating virulence	Virion
pC129R	15	*C129R*	Evasion from host defense	Virion
pEP153R	18.4	*EP153R*	C-type lection-like protein, evasion from host defense	Virion
pB169L	18.8	*B169L*	Unknow, transmembrane domain	Virion
pH171R	20	*H171R*	Unknow	Virion
pCP312R	35.2	*CP312R*	Unknow	Virion
pC257L	29.7	*C257L*	Unknow, transmembrane domain	Virion
pE146L	16.3	*E146L*	Unknow, transmembrane domain	Virion
pC717R	84.1	*C717R*	Unknow	Virion
pEP152R	17.8	*EP152R*	Unknow, transmembrane domain	Virion
pK421R	49.7	*K421R*	Unknow	Virion
pK145R	17.2	*K145R*	Unknow	Virion
pK196R	22.4	*K196R*	Genome replication, repair or transcription	Nonstructural
pB475L	56.1	*B475L*	Unknow	Nonstructural
pB602L	61.3	*B602L*	Promote proper folding of p72 protein	Nonstructural
pB125R	14.8	*B125R*	Unknow	Nonstructural
pK205R	23.7	*K205R*	Autophagy related	Nonstructural

### 3.2. Dominant antigens of ASFV

**Figure 1:**
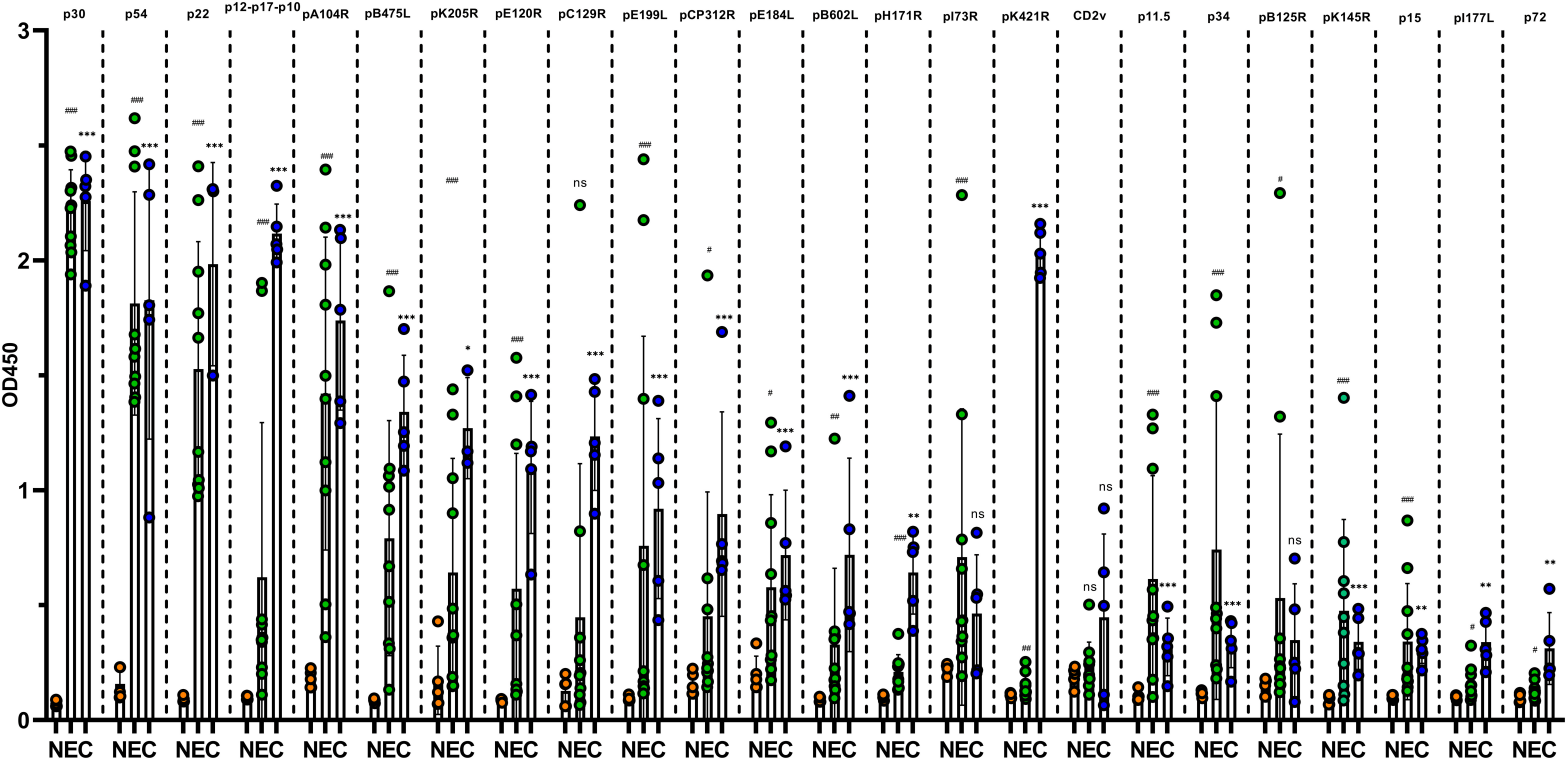
Reaction of ASFV recombinant proteins with antibodies in pig sera. ELISAs based on purified ASFV proteins were constructed and used to test five clinical ASFV-positive sera from farm pigs (C group, blue dot), 10 ASFV-positive pig sera experimentally infected with low-virulence ASFV (E group, green dot) and five ASFV-negative pig sera (N group, orange). The data of each group are presented as means and SD and were tested for significance using Student's *t*-test. * indicates a significant difference between the C group and N group (*, *P* < 0.05; **, *P* < 0.01; and ***, *P* < 0.001). ^#^ indicates a significant difference between the E group and N group (^#^, *P* < 0.05; ^##^, *P* < 0.01; and ^###^, *P* < 0.001).

A total of 15 positive pig sera for anti-ASFV antibodies (10 from pigs experimentally infected with attenuated ASFV and five from clinical field samples) and five pig sera free of ASFV were detected with ELISA plates coated with ASFV proteins. As shown in Figure 1, 24 proteins reacted with ASFV sera in varying degrees. p30, p54, and p22 showed a positive reaction with all ASFV sera (the value of sample/negative ≥3), which suggested that they were the dominant antigens of ASFV. pA104R, pB475L, pE120R, pC129R, pE199L, pCP312R, pE184L, pB602L, pK205R, pH171R, and the fusion protein p12-p17-p10 showed a positive reaction with all five clinical ASFV sera and with only some of the experimentally infected ASFV sera. p34, p11.5, pB125R, pK145R, CD2v, pK421R, p15, pI177L, p72, and fusion protein pI73R-pB169L showed a positive reaction with some of the clinical and experimentally infected ASFV sera. The other 11 proteins showed a negative reaction with all ASFV sera (Supplementary material S3). These results indicated that some ASFV proteins such as p30, p54, and p22 have excellent antigenicity and stimulate strong immune responses. Some viral proteins, however, did not induce any antibody immune responses in ASFV infection.

### 3.3. ASFV induced rapid and strong p30-antibody response in infected pigs

**Figure 2:**
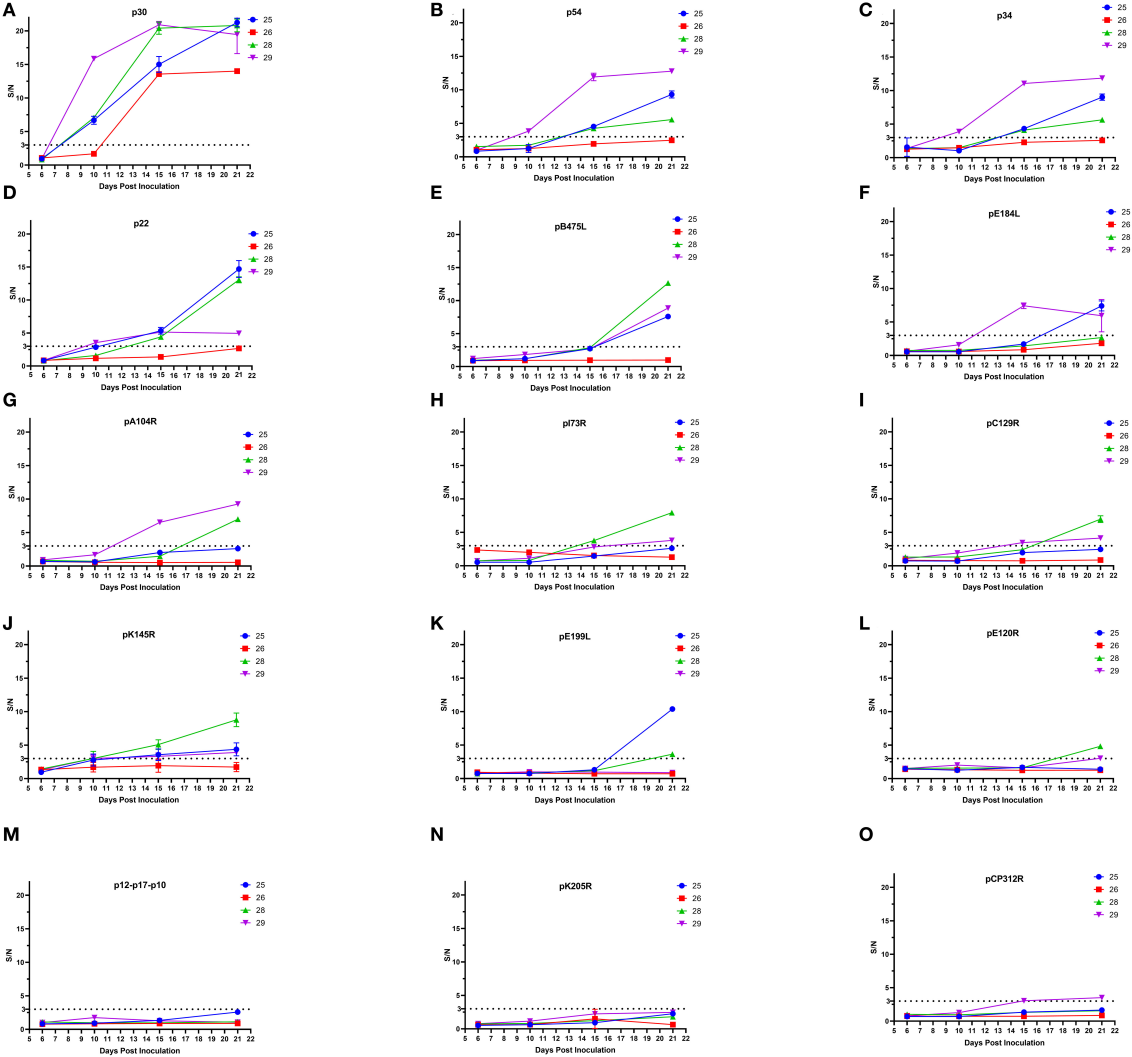
Development of ASFV-specific antibodies in pigs infected with low-virulence ASFV. ELISA methods based on 15 ASFV recombinant proteins including p30 **(A)**, p54 **(B)**, p34 **(C)**, p22 **(D)**, pB475L **(E)**, pE184L **(F)**, pA104R **(G)**, pI73R-pB169L **(H)**, pC129R **(I)**, pK145R **(J)**, pE199L **(K)**, pE120R **(L)**, p12-p17-p10 **(M)**, pK205R **(N)**, and pCP312R **(O)** were used to test the sera of four pigs (the number 25, 26, 28, and 29) at 6, 10, 15, and 21 dpi with low-virulence ASFV. A total of five ASFV-negative pig sera served as negative controls. If S/N≥3 (cutoff value), the sample was positive for antibodies against ASFV protein.

Some ASFV proteins were able to induce host antibody immunity. To further explore the properties of antibody generation during ASFV infection, pig sera were collected at 6, 10, 15, and 21 days after attenuated ASFV infection, and levels of antibodies against 15 ASFV proteins of high antigenicity were detected by ELISA. As shown in Figure 2, three of four pigs showed p30-antibody positive conversion (S/N ≥3) at 10 dpi. All four pig sera were p30-antibody positive, and S/N for p30 antibodies was >10 at 15 dpi. A total of three pigs became antibody positive for p22, p34, and p54 at 15 dpi and for pB475L and pK145R at 21 dpi, but the level of antibodies against these proteins remained negative in the rest of one pig. In total, one to two pigs developed antibodies against pA104R, pE184L, pC129R, pE199L, pE120R, and pCP312R and the fusion protein pI73R-pB169L. These results showed that attenuated ASFV infection induced a rapid and strong p30-antibody response.

## 4. Discussion

African swine fever virus (ASFV) is a highly complex enveloped DNA virus and encodes >150 ORFs (3). At present, it has been confirmed that ~100 proteins are expressed by ASFV, and 63 proteins form virion (8, 9). However, the roles of many structure proteins are uncharacterized. Clarifying the antigenicity of ASFV proteins is important to develop serological diagnostic methods and vaccines against ASFV. In this study, 33 genes of structural proteins with high abundance in virion and five genes of non-structure proteins with high expressing levels in host cells were expressed by *E. coli* to screen the dominant antigens of ASFV (8, 9). Among 33 structure proteins, many proteins are important components of the virion and play an important role in virus entry and morphogenesis, and the others are uncharacterized (Table 1). Indirect ELISAs based on these proteins were established to detect the antibody levels in the sera of pigs infected by ASFV. It was confirmed that ASFV infection induced strong antibody responses against p30, p54, and p22. pA104R, pB475L, p34, pE120R, pC129R, pE199L, pE184L, pCP312R, pK145R, and pB602L also induced distinct antibodies.

In this study, ASFV proteins were prepared using an *E. coli* expression system. To improve the expression levels of ASFV proteins in *E. coli*, the gene sequences were optimized. The signal peptides and transmembrane domains of proteins were removed. For several large ASFV proteins including pM1249L, pB602L, pCP717R, and p72, the predicted antigen dominant regions were expressed. Protein truncation may reduce detecting the special antibodies against natural proteins. Reis et al. showed strong antibody responses against pB602L (15). In our study, most sera from pigs infected with attenuated ASFV showed a weak reaction with the truncated pB602L protein. The genotype of ASFV may affect the generation of special antibodies. The sera from clinical samples in our study showed a stronger reaction with the truncated pB602L protein than the sera from the pigs infected with attenuated ASFV. The p72 protein is the major capsid protein and one of the most immunogenic ASFV proteins, which is an important target for test and vaccine development (16–19). In our study, however, the expressed p72 showed poor reactivity with the ASFV-positive sera, which may be because the p72 protein did not form the correct structure. It has been shown that correctly folded p72 needed the assistance of pB602L (20). Thus, the p72 protein which was expressed alone in *E. coli* may not be a suitable diagnostic antigen.

Among 35 ASFV proteins expressed in this study, p30, p54, and p22 were the dominant antigens during ASFV infection, which was consistent with previous studies (15, 17). To improve the sensitivity and specificity of p30 and p54-based ELISAs, most p30 and p54 were produced in insect cells using a baculovirus expression system (21). Although p30 protein was expressed in inclusion bodies in *E. coli* in our study, refolded p30 protein showed a strong reaction with all ASFV-positive sera. The p30 antibody developed early during attenuated ASFV infection and was the most sensitive target detected by ELISA. At 10 dpi, p30 antibodies became positive in most pigs. Even in one pig that remained negative for antibodies against other ASFV proteins, p30 antibodies became positive at 15 dpi. The indirect immunoperoxidase test is the best for ASF serological diagnosis due to its superior sensitivity. ASFV antibodies detected with immunoperoxidase became positive at 8–10 dpi in subacute or chronic infection, which is consistent with the seroconversion time of the p30 antibody in our study (22, 23). Therefore, the p30 antibody was an excellent target for ASF serodiagnosis. The p30 protein expressed in *E. coli* was also suitable as a diagnostic antigen for ELISA.

At present, there is no safe and effective vaccine available against ASFV (24). Several subunit vaccines including DNA- and peptide-based vaccines based on some major antigens, such as p30, p54, p72, CD2v, and/or pEP153R, provide partial protection against virulent ASFV challenge (25, 26). In addition to p54, p30, and hemagglutinin, Lacasta et al. suggested that some unidentified protective determinants within the ASFV genome could play an important role in preventing ASFV infection (13). Combining the results of ASFV proteins reacting with ASFV-positive sera and the development of ASFV antibodies in this study, pB475L, pC129R, pE199L, pE184L, and pK145R induced rapid antibody development in ASFV infection, besides p30, p54, and p22. pB475L is a non-structural protein, while pC129R, pE199L, pE184L, and pK145R are structural proteins. E199L can mediate virus entry but the other four proteins are uncharacterized (27). It is unclear whether these five proteins can induce protective immunity against ASFV.

## 5. Conclusion

In this study, p30, p54, and p22 were identified as the dominant antigens of ASFV. In particular, the development of the p30 antibody was rapid and strong during ASFV infection. Thus, it was an important target of serodiagnosis. pB475L, pC129R, pE199L, pE184L, and pK145R also induced antibody development in ASFV infection. These results promoted the development of subunit vaccines and serodiagnostic methods against ASFV.

## Data availability statement

The raw data supporting the conclusions of this article will be made available by the authors, without undue reservation.

## Author contributions

ZX and YH: methodology, investigation, and validation. JL, AW, and XM: formal analysis and validation. LC: investigation. JW, WT, LY, NK, and HY: methodology and resources. TS: review and editing. GT: project administration. GW: supervision and writing—review and editing. HZ: conceptualization, methodology, writing–original draft, supervision, and funding acquisition. All authors have read and agreed to the published version of the manuscript.
