# Supplementary material for: Screening and identification of the dominant antigens of the African swine fever virus
Source: Front Vet Sci. 2023 May 4;10:1175701. doi: 10.3389/fvets.2023.1175701 (PMC10192620; doi:10.3389/fvets.2023.1175701)
Supplement: Supplementary file 1 [file Data_Sheet_1.pdf]

S1. The amino acid sequences of ASFV proteins expressed by E. coli

>p15NVIHADLHLNNMTYYHFNPTSFTDRNKPGKYTLKVKNPVIAFITGPKVETETYVFKHIDGFGCIID
FSRAIMGPNHAIKLERQYGLAFVNTFYRNQSEHILKVLRYYFPEMLTNRENEIQGVILSNFNFFFNSITAI
DFYAIARNLRSMLSLDYLHTSEVKRNVEISQTFLDTCQFLEEKAVEFLFKNLHTVLSGKMPSNMKQFCK
ISVWLQQHDPDLLEIINNLCMLGNLSAAKYKHGVTFIYPKQAKIRDEIKKHAYSNDPSQAIKTLESLILPF
YIPTPAEFTGEIGSYTGVKLEVEKTEANKVILKNGEAVLVPAADFKPFPDRRLAVWIMESGSMPLEGPPY
KRKKEGG

>p34
MGDKNPVQHIKDYHIDSVSSKAKLRIIEGIIRAIAKIGFKVDTKQPIEDILKDIKKQLPDPRAGSTFVKNAE
KQETVCKMIADAINQEFIDLGQDKLIDTTEGAASICRQIVLYINSLTHGLRAEYLDVHGSIENTLENIKLL
NDAIKQLHERMVTEVTKAAPNEEVINAVTMIEAVYRRLLNEQNLQINILTNFIDNILTPTQKELDKLQT
DEVDIIKLLNDTNSVLGTKNFGKVLS

>E120R
MADFNSPIQYLKEDSRDRTSIGSLEYDENADTMIPSFAAGLEEFEPIPDYDPTTSTSLYSQLTHNMEKIA
EEEDSNFLHDTREFTSLVPDEADNKPEDDEESGAKPKKKKHLFPKLSSHKSK

>A137R
MEAVLTKLDQEEKKALQNFHRCAWEETKNIINDFLEIPEERCTYKFNSYTKKMELLFTPEFHTAWHEVP
ECREFILNFLRLISGHRVVLKGPTFVFTKEIKNLGIPSTINVDFQANIENMDDLQKGNLIGKMNIKEG

>I73R-B169L
METQKLISMVKEALEKYQYPLTAKNIKVVIQKEHNVVLPTGSINSILYSNSELFEKIDKTNTIYPPLWIRKN
AEAAAKEAAAKANHQLNDIYNKSNMDVIVSSIHDKYKGGDEIIPPISPPSVSNELEEDQPKKIPAGPKP
ADSKPVSLPDSKPLVPLQEVIMPSQYNN

>p12-p17-p10
MPRQQKKCSKAEECTCNNGSCSLKTSAEAAAKEAAAKANRTIDCKSSIPKPPPSYYVQQPEPHHHFP
VFFRKRKNSTSLQSHIPSDEQLAELAHSAEAAAKEAAAKAMPTKAGTKSTANKKTTKGSSKSGSSRGH
TGKTHASSSMHSGMLYKDMVNIARSRGIPIYQNGSRLTKSELEKKIKRSK

>H240R
MAANIIATRAVPKMASKKEHQYCLLDSQEKRHGHYPFSFELKPYGQTGANIIGVQGSLTHVIKMTVFP
FMIPFPLQKTHIDDFIGGRIYLFFKELDMQAVSDVNGMQYHFEFKVVPVSPNQVELLPVNNKYKFTYAI
PVVQYLTPIFYDLSGPLDFPLDTLSVHVDILSNHIQLPIQNHNLTTGDRVFISGYKHLQTIELCKNNKIFIK
NIPPLSSEKIKLYILKNRIRIPLYFKSLKTSK

>EP153R
MEINKPICYQNDDKIFYCPKDWVGYNNVCYYFGNEEKNYNNASNYCKQLNSTLTNNNTILVNLTKTL
NLTKTYNHESNYWVNYSLIKNESVLLRDSGYYKKQKHVSLLYICSK

>C129R
MEHPSTNYTPEQQHEKLKHYVLIPKHLWSYIKYGTHVRYYTTQNVFRVGGFVLQNPYEAVIKNEVKTA
IRLQNSFNTKAKGHVTWAVPYDNISKLYAKPDAIMLTIQENVEKALHALNQNVLTLASKIR

>A104R
MSTKKKPTITKQELYSLVAADTQLNKALIERIFTSQQKIIQNALKHNQEVIIPPGIKFTVVTVKAKPARQG
HNPATGEPIQIKAKPEHKAVKIRALKPVHDMLN

>B125R
MAVYAKDLDNNKELNQKLINDQLKIIDTLLLAEKKNFLVYELPAPFDFSSGDPLASQRDIYYAIIKSLEER
GFTVKICMKGDRALLFITWKKIQSIEINKKEEYLRMHFIQDEEKAFYCKFLESR

>K145R

MDHYLKKLQDIYTKLEGHPFLFSPSKTNEKEFITLLNQALASTQLYRSIQQLFLTMYKLDPIGFINYIKTSK
QEYLCLLINPKLVTKFLKITSFKIYINFRLKTFYISPNKYNNFYTAPSEEKTNHLLKEEKTWAKIVEEGGEES
>p150
MLGDAQNNTSDVVRKRLVAVIDGIIRGSHTLADSAMEVLHELTDHPIYLETEEHFIQNYMSRYNKEPL
MPFSLSLYYLHDLRIENNEVYDPLLYPNLESGSPEFKLLYGTRKLLGNDPVQLSDMPGVQLIMKNYNET
VVAREQITPTRFEHFYTHAIQALRFIINIRSFKTVMMYNENTFGGVNLISENRDDKPIITAGIGMNAVYS
LRKTLQDVISFVESSYQEEQINHIHKIVSPKGQTRTLGSNRERERIFNLF
>M1249L
MIPMHKPRTPKEAEYEFKTVIGRTPAELLSQKEFYDKIYTSKYRPDFTQLTRLNDIYFQEESLRVWWGGR
DEEKTSTLIYLRAYELFLKYLQNAPNFNSELAEFKTYENAYGEQKALLAQQGFYNIFDPNTGRADQRTR
LFEYKRLPISTLYDERGLPHKWTIYVYKAVDSSQKPAEIEVTRKDVIKKIDNHYALADLRCSVCHVLQHE
VGQLNIKKVQTALKASLEFNTFYAFYESRCPKGGLHDFQDKKCVKCGLFTYIIYDHLSQPELVHDYYNN
YKDQYDKEKMSIRSIQ
>E184L
MKTFITCTSVKNYFRQHLKTNQRISSELISYVCTILNHICHQYLQNPQAQEEEWFALIKELPIIKDGLSKE
ERFFSSGVKHFLHEYKITPENQEKFQKMLNAITEQLMSRLCKVFSIMIQRQGFLKTQTLMYSHLFTILSIL
MVADNLYGEQDPTEFFSLIIEQTKTIKKKKKSGSEEEESHEE
>H171R
MVVYDLLVSLSKESIDVLRFVEANLAAFNQQYIFFNIQRKNSITTPLLITPQQEKISQIVEFLMDEYNKN
NRRPSGPPREQPMHPLLPYQQSSDEQPMMPYQQPPGNDDQPYEQIYHKKHASQQVNTELNDYY
QHILALGDEDKGMDSMLKLPEKAKRDSDDEDDMFSIKN
>B475L
MHKVTKEMQDYSLTFLLKKRMELYNKFLRKQAYVEPETELEETYARLSSYNRSLLHQIEELTSENKSLLA
DLSTLRKKYEKRQSEYRRLVQLLYQQIQRSSTSKSSYPLTKFIETLPSEHFSNEEYQKETPADQKEVVEME
LLRKQELLTSQELTSKSPNNYPVPHSRTIVSKPLDNYPVPRSRTTTKIDFDNSLQNQELHTKNGFSEKDI
VEFGQDKPEEENILAIDQDKPEEETILAIKQDISEEDNIFAIDQDKPEFNQDTPEFKEAVLDIKENILEEEN
QDEPIVQNPFLENFWKPEQKTFNQSGLFEESSNFSNDWSGGDVTLNFS
>CP312R
MTTHIFHADDLLQALQQAKAEKNFSSVFSLDWDKLRTAKRNTTVKYVTVNVIVKGKKAPLMFNFQN
EKHVGTIPPSTDEEVIRMNAENPKFLVKKRDRDPCLQFNKYKISPPLEDDGLTVKKNEQGEEIYPGDEE
KSKLFQIIELLEEAFEDAVQKGPEAMKTKHVIKLIQRKISNSAVKNADKPLPNPIARIRIKINPATSILTPILL
DKNKPITLQNGKTSFEELKDEDGVKANPDNIHKLIESHSIHDGIINARSIC
>C717R
MDLCYGAYVLHKKENVIHADLHLNNMTYYHFNPTSFTDRNKPGKYTLKVKNPVIAFITGPKVETETYV
FKHIDGFGCIIDFSRAIMGPNHAIKLERQYGLAFVNTFYRNQSEHILKVLRYYFPEMLTNRENEIQGVILS
NFNFFFNSITAIDFYAIARNLRSMLSLDYLHTSEVKRNVEISQTFLDTCQFLEEKAVEFLFKNLHTVLSGK
PVEKTAGDVLLPIVFKKFLYPNIPKNILRSFTVIDVYNYNNIKRYSGKAIQTFPPWAQTKEILTHAEGRTFE
DIFPRGELVFKKAYAENNHLDKILQRIREQLANENL
>F317L
MVETQMDKLGFLLNHIGKQVTTKVLSNAHITQTMKEIILENHSVDGGAAKNVSKGKSSPKEKKHWTE
FESWEQLSKSKRSFKEYWAERNEIVNTLLLNWDNVRGAIKKFLDDDREWCGRINMINGVPEIVEIIPSP
YRAGENIYFGSEAMMPADIYSRVANKPAMFVFHTHPNLGSCCGGMPSICDISTTLRYLLMGWTAGH
LIISSNQVGMLTVDKRIIVDLWANENPRWLMAQKILDIFMMLTSRRSLVNPWTLRDLKKILQDYGIEYII
FPSNDFFIYEDERLLMFSKKWTNFFTLHELLDDLETIETKASSTT

>K196R
MERLEKKVVFIKSTKNTRDKTIKTHSGIQLRPKQCKIIESTQLSDVGSLTDIHAVVVDEAHFFDDLITCRT
WAEEEKIIILAGLNASFEQKMFPPIVRIFPYCSWVKYIGRTCMKCNQHNACFNVRKNADKTLILAGGSE
LYVTCCNNCLKNTFIKQLQPIKY
>K421R
MYTHVDVVGIAEASAALYVQKDRDRYLDVLTTIENFIYQHKCIITGESAHLLFLKKNIYLYEFYSNNVAE
HSKALATLLYKLDPEYLTRYTVLITKIPNHWYVINVDQREFVRLYAIPAVKQHLPIPILPFYCTSALTQQEL
FCLGPELQLIQIYSKLCNPNFVEEWPTLLDYEKSMRMLFLEQFPQRLEMTGGKKEEKEKHESIIKKIILEM
VSTRQRIVVGGYIQKNLYNHVLKNRNRLQLITSLNIYEEKDIIQQFCDSNGLKIKIRINNPLLPTNPELRR
LTIYFNHNNDDDQSYLIVDMYNTGSYELVPTNQINT
>B602L
MYEELRAATESIYPEKPDLEFAFIIYDVVDSSNQQKVDEFYYKYKDQIFSEVSSIQLGNWTLLGSFKANR
ERYNYFNQNNEIIKRILDRHEEDLKIGKEILRNTIYHKKAKNIQETGPDAPGLSIYNSTFHTDSGIKGLLSF
KELKNLEKASGNIKKAREYDFIDDCEEKIKQLLSKENLTPDEESELIKTKKQLDNALEML
>C257L
MYSVCDVVRDAVAQSHLCACPNDKLPQCKGVTKAPPKCSVFHVAKLQDTKFKWKYTLDPLKAQKLS
QIDKDIEKDAITLKLIYGIELSPEDLEWWKMQRCLINKKTGAKGGQFANKYLERQDLELLGYSPTPIIGG
DFMFTALPDKVLRTIPVAWDRFLN
>E199L
MSCMPVSTKCNDIWVDFSCTGPSISELQKKEPKAWAAILRSHTNQQTAEDDNIIGSICDKQGLCSKD
EYAYSQYCACVNSGTLWAECAFAPCNGNKNAYKTTEQRNILTNKQCPSGLTICQNIAEYGGSGNISD
LYQNFNCNSVINT
>K205R
MVEPREQFFQDLLSAVDQQMDTVKNDIKDIMKEKTSFMVSFENFIERYDTMEKNIQDLQNKYEEMA
ANLMTVMTDTKIQLGAIIAQLEILMINGTPLPAKKTTIKEAMPLPSSNTNNEQTSPPASGKTSETPKKN
PTNAMFFTRSEWASSNTFREKFLTPEIQAILDEQFANKTGIERLHAEGLYMWRTQFSDEQKKMVKEM
MKK
>p22
MKKQQPPKKVCKVDKDCGSGEHCVRGSCSSLSCLDAVKMDKRNIKIDSKISSCEFTPNFYRFTDTAAD
EQQEFGKTRHPIKITPSPSESHSPQEVCEKYCSWGTDDCTGWEYVGDEKEGTCYVYNNPHHPVLKYG
KDHIIALPRNHKHA
>E146L
MGWSPFKYSKGNTVTFKTPDESSIAYMRFRNCVFTFTDPKGSLHSIDVTEVLNNMAKGFRDAQNPPS
SFTLGGHCQAPLNAFSFVLPGVNDRATVATADEAKKWENCDATLTGLQRII
>E248R
MGGSTSKNSFKNTTNIISNSIFNQMQSCISMLDGKNYIGVFGDGNILNHVFQDLNLSLNTSCVQKHV
NEENFITNLSNQITQNLKDQEVALTQWMDAGTHDQKTDIEENIKVNLTTTLIQNCVSSLSGMNVLVV
KGNGNIVENATQKQSQQIISNCLQGSKQAIDTTTGITNTVNQYSHYTSKNFFDFIADAISAVFK
>EP152R
MRADHARKYLEGMWHGDPVFLKQSGLQSFYLYIQPDHTCFFSIVNKNGEKLMETKIPCTITNKIYMFF
KPIFEFHVVMEDIHSYFPKQFNFLLDSTEGKLILENNHVIYAVLYKDNFATALGKTVEKYITQN
>I177L
MKTPFKCITTTKTPVLFIKFQLIAADNYQAITWKDGILNYEKIDQPTPLYLSVNGLIFDCAKLQPLTTKSN
VTSGDKVVHIGQTFEYNNLLMWKVNDQGFLNISVTGTKFNLIAITGKLGFYTDPPSHLIIMPLKFFPVH

KFSKNEPNKKQKRFIYF
>p30
MDFILNISMKMEVIFKTDLRSSSQVVFHAGSLYNWFSVEIINSGRIVTTAIKTLLSTVKYDIVKSARIYAG
QGYTEHQAQEEWNMILHVLFEEETESSASSENIHEKNDNETNECTSSFETLFEQEPSSEVPKDSKLYML
AQKTVQHIEQYGKAPDFNKVIRAHNFIQTIYGTPLKEEEKEVVRLMVIKLLKKK
>p54
MSSRKKKAAAIEEEDIQFINPYQDQQWVEVTPQPGTSKPAGATTASVGKPVTGRPATNRPATNKPVT
DNPVTDRLVMATGGPAAAPAAASAPAHPAEPYTTVTTQNTASQTMSAIENLRQRNTYTHKDLENSL
>CD2v
MNITNDNNDINGVSWNFFNNSFNTLATCGKAGNFCECSNYSTSIYNITNNCSLTIFPHNDVFDTTYQ
VVWNQIINYTIKLLTPATPPNITYNCTNFLITCKKNNGTNTNIYLNINDTFVKYTNESILEYNWNNSNIN
NFTATCIINNTISTSNETTLINCTYLTLSSNYFYTFF
>p72
MPEIHNLFVKRVRFSLIRVHKTQVTHTNNNHHDEKLMSALKWPIEYMFIGLKPTWNISDQNPHQHR
DWHKFGHVVNAIMQPTHHAEISFQDRDTALPDACSSISDISPVTYPITLPIIKNISVTAHGINLIDKFPSK
FCSSYIPFHYGGNAIKTPDDPGAMMITFALKPREEYQPSGHINVSRAREFYISWDTDYVGSITTADLVV
SASAINFLLLQNGSAVLRYST

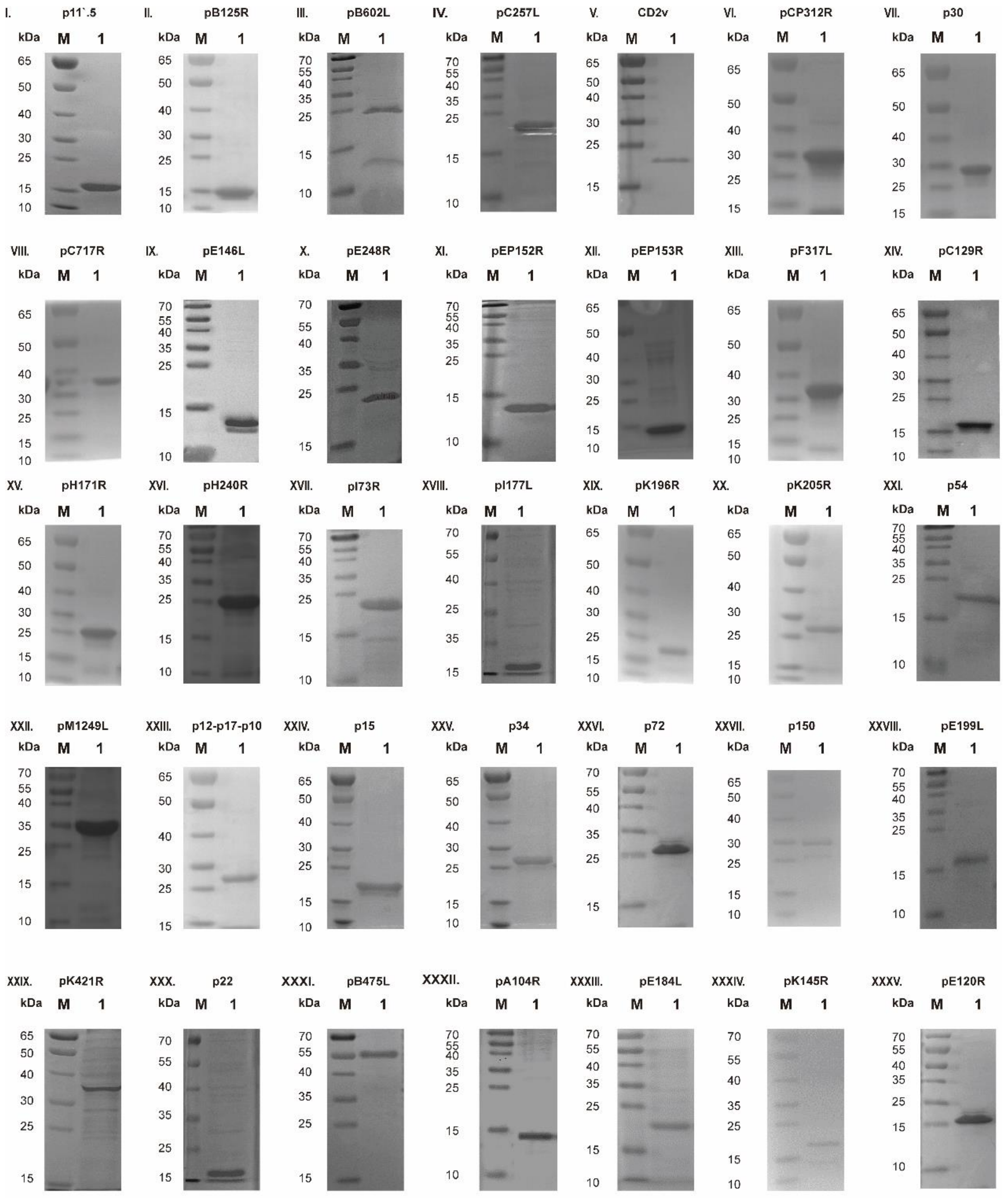


S2. Purification of ASFV proteins. ASFV proteins were expressed by pCold I in E. coli and then purified with WorkBeads 40 Ni-NTA.

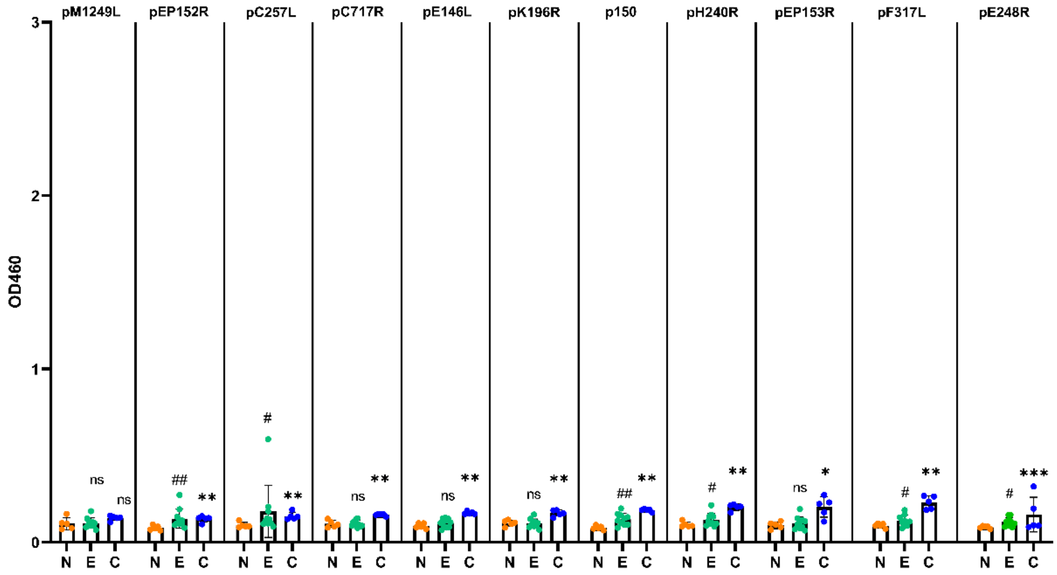


S3. Reaction of 11 ASFV recombinant proteins with antibodies in pig sera. ELISAs based on 11 purified ASFV proteins were constructed and used to test five clinical ASFV-positive sera from farm pigs (C group), 10 ASFV-positive pig sera experimentally infected with low-virulence ASFV (E group) and five ASFV-negative pig sera (N group). The data of each group are presented as means and SD and were tested for significance using Student's t test. * indicates a significant difference between C group and N group (*, P < 0.05; **, P < 0.01; ***, P < 0.001). # indicates a significant difference between E group and N group (#, P < 0.05; ##, P < 0.01).
